# Parkinson’s disease and awareness of the passage of time: Perceived health, emotion, and self-transcendence

**DOI:** 10.1371/journal.pone.0352176

**Published:** 2026-07-17

**Authors:** Florie Monier, Sylvie Droit-Volet, Michael Dambrun, Sophie Monceau, Laure Delaby, Isabelle Rieu, Tiphaine Vidal, Franck Durif, Ana Marques

**Affiliations:** 1 Université Clermont Auvergne, CNRS, UMR 6024, Clermont-Ferrand, France; 2 Centre Hospitalier Universitaire de Clermont-Ferrand, Clermont-Ferrand, France; Gabriele d’Annunzio University of Chieti and Pescara: Universita degli Studi Gabriele d’Annunzio Chieti Pescara, ITALY

## Abstract

This study examined passage-of-time judgments in patients with Parkinson’s disease, compared to age-matched healthy controls. The aim was to determine whether these temporal judgments are influenced by affects or by psychological representations of the self, which may differ between these populations. The self has indeed been identified as a potential factor shaping time cognition, either through the minimal self, rooted in emotional and bodily sensations, or through cognitive self-representations. We used self-reported scales to measure participants’ passage-of-time judgments across two distinct dimensions: (1) for distant life periods (e.g., now compared to 5 years ago), and (2) for shorter and more recent time periods (e.g., day, week). Participants also completed questionnaires assessing emotional states (e.g., perceived health, well-being, happiness). To more specifically explore self-representation, we measured levels of self-transcendence, defined as the pursuit of meaning that extends beyond oneself and one’s own needs or interests, to examine whether individuals viewed themselves in a self-centered way or held a more open and interconnected sense of self. The results did not show that Parkinson’s disease alter passage-of-time judgments, whether related to recent or distant past periods. However, the findings confirmed that judgments about the distant past and recent experiences reflect two distinct forms of temporal judgment. The former was associated with self-consciousness and the projection of the self across the lifespan, whereas the latter is more closely linked to emotional and bodily state representations, shaped by perceived health, emotional condition, and overall well-being.

## Introduction

In recent years, there has been a growing interest in the feeling of the passage of time (PoT), with an increasing number of studies conducted on typically developing children, adults, and older adults. However, PoT judgments in clinical populations remain largely unexplored. Parkinson’s disease, characterized by impairments in the basal ganglia, is well known to alter duration perception [1–7]. We thus chose to examine PoT judgments, a new aspect of temporal processing, in patients with Parkinson’s disease (PD), compared to age- and sex-matched control participants. This type of research is of dual interest. On the one hand, it refines our understanding of the mechanisms underlying PoT judgments. On the other hand, it may shed light on the psychological consequences of neurodegenerative diseases on time cognition, extending beyond duration perception. Such insight should contribute to the development of more effective clinical approaches for these pathologies [8].

PoT judgments refer to the phenomenological experience of time elapsing and represent a specific form of temporal awareness related to the projection of the self into a temporal perspective [9,10]. Different types of PoT judgments have been described depending on the time span considered [10,11]. Some are associated with the projection of the self across long periods, encompassing the entire lifespan—for example, the reported feeling that time accelerates with aging, or when comparing the current experience of time passage with that of years past (e.g., now compared to before, or to 5–10 years ago). Others are related to the experience of time over shorter and more recent periods, such as the interval that has just ended. This type of PoT is typically assessed when individuals are asked about their perception of PoT during the day, the past week, or year. Finally, present PoT judgments refer to the immediate, ongoing feeling of time passing [12–15].

Recent studies have examined the psychological processes involved in PoT judgments and have highlighted distinct patterns of underlying factors, depending on whether PoT refers to long past periods of life or to shorter intervals, including the present. By investigating present PoT judgments, Martinelli and Droit-Volet [15,16] demonstrated that the PoT judgments for short and recent intervals are strongly influenced by emotional factors depending on context, and proposed the Contextual Self-Duration Theory [9,10,15,16]. According to this theory, these PoT judgments are determined by individuals’ awareness of their internal states, namely the emotional and bodily sensations experienced in a given context. In particular, the ability to read bodily information associated with emotions, i.e., interoceptive awareness, has been shown to be a key ability in detecting changes in the passage of time [14,17–20]. The current feeling that time is slowing down therefore mainly depends on emotions of sadness and boredom, as shown in numerous studies on PoT judgments during the COVID-19 lockdowns [21–23]. In contrast, the feeling that time passes quickly is associated with happiness and well-being. Ultimately, the predominant factor regulating variations in the feeling of time passage over short periods—including the present, week, month, and year—appears to be related to an individual’s capacity to remain aware of bodily and emotional experiences [14,20,24].

In contrast, retrospective judgments about the passage of time over long past periods of life (e.g., now compared to 5 years ago, now compared to before, or across aging) appear to be more closely related to the narrative self than to the minimal self [10]. The narrative self refers to the general knowledge of oneself in its temporality, encompassing memories of the past and intentions for the future—that is, a conceptual self detached from immediate, everyday experience [25,26]. The feeling that time accelerates with age thus stems from projecting oneself into a future known to be limited, while the past continues to expand. This awareness of life’s finiteness leads individuals to believe that time passes faster as they grow older, particularly after the age of 50 [27,28]. However, although older adults often report that time seems to pass more quickly with aging, they do not actually experience a faster present passage of time compared to younger adults [12]. By contrast, individuals over the age of 75, especially those living in nursing homes, frequently report a slowing down of time [29]. This finding is consistent with studies showing no correlation between present PoT judgments and retrospective PoT judgments over long life periods [12,30].

There is therefore no significant correlation between PoT judgments for short periods and long life periods, as they rely on distinct underlying processes. Nevertheless, both types of judgments are grounded in the self. We thus sought to explore whether judgments of the passage of time might be related to one’s stance toward the importance of the self, specifically an individual’s level of selflessness, i.e., self-transcendence. The concept of self-transcendence refers to the capacity of human beings to “be directed by something other than themselves” [31], and to remain open to others and to the world as part of a broader whole. In other words, self-transcendence involves in moving beyond one’s immediate personal interests and needs to focus on something greater than oneself: a cause, values, others, an ideal, or a spiritual or collective dimension. An individual with Parkinson’s disease may experience a sense of self-transcendence by finding meaning beyond their illness, for example, by helping others with the same disease, strengthening their relationships with loved ones, or focusing on their spiritual and personal growth despite their physical limitations. The self-transcendence is closely linked to self-detachment or selflessness. By contrast, self-centeredness gives rise to the pursuit of ego-related satisfactions, which are frequently threatened by frustrations, thereby fostering fluctuating happiness and afflictive emotions. Conversely, as argued by Dambrun and Ricard [32], selflessness—closely associated with self-transcendence—promotes a deeper and more stable form of happiness. Numerous studies have further shown that selflessness is associated with reduced psychological distress [33], greater well-being [34,35], and more stable positive affects [32,36–38]. Accordingly, we hypothesized that self-transcendence could be a significant predictor of variations in PoT judgments across both short periods (e.g., present, day, week) and longer life spans (e.g., 5 years ago, now compared to before, with aging).

Over short periods, it is reasonable to assume that a high level of self-transcendence, which fosters a stable state of happiness, may reduce the feeling that time is slowing down. Over longer periods, however, it becomes more difficult to predict whether PoT will accelerate or decelerate with self-transcendence. Two distinct mechanisms may account for this influence: a psychological defensive mechanism and a cognitive autobiographical mechanism. Regarding the defensive mechanism, several studies have shown that self-transcendence reduces death anxiety [39–41]. Ego-related threats such as aging or death may thus trigger a deceleration of PoT through protective strategies that mentally delay feared events—namely, by slowing down the subjective course of life leading to death. Conversely, higher levels of self-transcendence, associated with reduced death anxiety, may enable individuals to accept the acceleration of lifetime progression toward death. As for the cognitive autobiographical mechanism, PoT over long intervals relies on self-narrative, particularly on the construction of temporal continuity between past, present, and future. Discontinuities in life narrative may become more salient when attention is focused on the self, leading to a subjective dilation of lifetime. In contrast, reducing the centrality of the self and personal events may enhance temporal fluidity and promote narrative compression. This interpretation is consistent with the effect of awe, which has been shown to produce a subjective acceleration of time [42]. In this study, we therefore examined a population of patients with an evolutive neurodegenerative pathology that is anxiety-inducing, although no direct question on death-related anxiety was asked for ethical reasons. This design allowed us to better investigate whether time perception is accelerated or decelerated over both short and long periods in relation to self-transcendence.

In this study, we aimed to further examine PoT judgments by comparing a typically aging population with patients diagnosed with Parkinson’s disease (PD). This neurodegenerative disorder primarily causes progressive damage to the basal ganglia [43]. One of its most consistent effects is a decline in motor skills and motor synchronization. Moreover, since the basal ganglia are strongly involved in interval timing via the dopaminergic system, PD patients also exhibit alterations in duration perception, especially for supra-second intervals [1,2,44,45]. It has been shown that PoT judgments are not based on the processing of temporal information per se (i.e., duration perception), but rather on the awareness of internal states (emotion, focusing attention on the task) [15,16]. Because PoT judgments rely on mechanisms distinct from duration-based timing, PD constitutes a theoretically informative model for examining how alterations in affective, interoceptive, and self-awareness processes shape PoT judgments. Accordingly, we hypothesized that Parkinson’s disease itself would not directly affect PoT judgments over short or long periods, but only through emotional and self-related processing differences between PD and Control participants.

Parkinson’s disease is known to alter interoceptive abilities [46,47] involves in the awareness of emotional states. In particular, interoceptive consciousness, i.e., the ability to read body information and felt emotions, is a key factor in PoT judgments [17]. Mioni et al. [48] showed that PD patients experienced fewer time distortions in response to emotional stimuli, particularly when exposed to emotional faces. According to these authors, this may reflect a deficit in the awareness of emotional and bodily signals, associated with impaired interoception. By examining present PoT judgment through the emergence of the feeling of the passage of time in an emotional context, Droit-Volet et al. [14] showed that participants with a high level of interoceptive awareness perceived more changes in the passage of time during a task than those with low interoceptive awareness. Participants with low interoceptive consciousness perceived no changes; their PoT judgments were based solely on a constant feeling of boredom that emerged very early in the task. As the passage of time seems to be strongly related to interoceptive awareness, we hypothesized that PD patients may present an alteration in their PoT judgments for short and recent periods. More specifically, as apathy and afflictive emotional states are key symptoms of PD [49–52], we expected that in the absence of accurate interoceptive consciousness, these afflictive affects would dominate the passage of time judgment, leading to a subjective feeling of time slowing down over short periods (present, days, weeks, and possibly the year), as this type of PoT judgment depends on awareness of emotional states.

There are few studies on self-transcendence and even fewer on differences between PD and control participants. Most studies have focused on other personality traits, indicating that PD are more introverted, anxious and cautions [53]. In addition, the personality of PD patients can change in the course of the disease and dopaminergic treatment leading to impulsivity and motor fluctuations [54]. Nevertheless, regarding self-transcendence, one study reported lower scores in PD patients [54] than in healthy individuals [55]. However, these results need to be verified, as demographic factors could account for this between-study difference. Indeed, the study by Pelisso et al. [55] included more women, and women generally score higher on self-transcendence than men. PD seems to alter the representation of the self [56], leading to a painful perception of one’s former self, reduced psychological distance, and increased anxiety toward the feared self [57]. Moreover, self-transcendence would serve as a strong protective factor against the anxiety induced by the perceived degradation of the self across time. This is why we assumed lower self-transcendence scores in PD than in control participants, as suggested by Boussac et al. [54]. Furthermore, as explained above, PoT judgments over long periods (e.g., now compared to 5 years ago, now compared to before, across aging) are based on self-narrative and projecting the self through time, whereas PoT judgments over short periods (e.g., present, week) are more influenced by emotions. We therefore hypothesized that self-transcendence scores would influence PoT judgments, with a greater effect on judgments over long periods than on those over short and recent periods.

In the present study, participants with PD and age- and sex-matched healthy controls completed a series of questions assessing different types of PoT judgments, perceived health, well-being, happiness, and serenity. We also evaluated whether their happiness was stable or fluctuating, as well as their levels of depression and anxiety. Finally, self-transcendence and mindfulness were assessed.

## Materials and methods

### Participants

The final sample consisted of 108 participants: 56 patients diagnosed with Parkinson’s disease (men = 40, women = 16, M_age_ = 65.37, SD_age_ = 6.12) and 52 healthy participants matched as closely as possible on age and sex (men = 32; women = 20, M_age_ = 66.09, SD_age_ = 7.56). The two groups did not differ significantly in age, *t*(106) = −0.547, *p* = .29, Cohen’s d = −0.105, and sex, *t*(104) = 1.08, *p* = .282, d = 0.209. PD patients were diagnosed with idiopathic Parkinson’s disease according to the criteria of the United Kingdom Parkinson’s Disease Brain Bank (UKPDBB) [58,59]. They were all receiving L-Dopa treatment. Based on exclusion criteria, none of the patients presented atypical parkinsonian syndrome (e.g., oculomotor disorders, cognitive impairment, apraxia, or early falls) [60]. Furthermore, they had no history of psychosis or progressive psychiatric disorders meeting DSM IV (Axis I), as well as schizotypal or schizoid personality disorders (Axis II). The absence of most cognitive impairments was ensured by a high score on the Montreal Cognitive Assessment (MoCA ≥ 24) [61]. More detailed information about PD patients is presented Table 1. They were recruited by the neuropsychology team at the neurology department of the Clermont-Ferrand University Hospital. Healthy controls were recruited from the entourage.

**Table 1:** Clinical information about patients with Parkinson’s disease (N= 56 patients).

	MEAN	SD
Disease duration (years)	8.39	4.13
UPDRS III ON/1 08	15.47	8.96
MOCA/ 30	26,36	2.62
Behavioural alterations/ 84^1^	8.05	5.44

^1^for the score calculation see Ardouin et al. (2009)

They all participated voluntarily in the study, with the option of not answering all questions and questionnaires. All participants were adults and were capable of providing informed consent in accordance with applicable ethical guidelines. Capacity to consent was determined by ensuring that participants were able to understand the study information, ask questions if needed, and voluntarily agree to participate prior to enrollment. No participants with known cognitive impairment or conditions affecting decision-making capacity were included in the study. In accordance with the guidelines of the French ethics committee, participants gave their oral consent first, and the questionnaire was then given to them. Consent was confirmed when participants returned the completed questionnaire. Despite their initial oral consent. The questionnaire was given to 27 additional individuals (27 patients) who ultimately declined to participate by not returning the questionnaire. The study protocol, including the consent procedure and the assessment of capacity to consent, was approved by the National Committee for the Protection of Persons WEST III (N°2018-A00840-55), and was performed in accordance with the principles of the Declaration of Helsinki.

### Procedure

Participants completed a series of 11 questions and 6 validated questionnaires among a set of other questionnaires, all presented in a paper booklet. Three different versions of the booklet were used varying the order of questionnaire presentation, while the 11 questions were always placed at the beginning.

Among the initial questions, 4 were emotion-related and addressed perceived health, general well-being, happiness, and serenity. Participants responded on a 7-point scale [see 12] ranging from 1 (“very low”) to 7 (“very high”). These questions were followed by 7 questions on PoT judgments. Four of them focused on short time periods: Do you think that time passes fast (1) now, (2) this week, (3) this month, (4) this year? The remaining three questions addressed longer time periods: “Do you think that time passes faster (1) now than 5 years ago, (2) now compared to before, and (3) with aging? Response scales were given on a 7-point scale ranging from 1 (“not at all”) to 7 (“a lot”). These different questions were used in numerous studies on PoT judgments [e.g., 11, 12, 27, 28]. The internal consistency of temporal items was high, as indicated by Cronbach’s Alpha (α = .914).

Participants then completed 5 questionnaires. (1) The Fluctuating Happiness Questionnaire was used to assess fluctuating happiness and (2) the Authentic Happiness Questionnaire to assess durable happiness [62]. The former consisted of 10 items rated on a 7-point scale from 1 (“strongly disagree”) to 7 (“totally agree”), while the latter consisted of 16 items rated from 1 (“very low”) to 7 (“very high”). (3) Depression levels were assessed with the Beck Depression Inventory (BDI) [63] and (4) anxiety levels with the State Trait Anxiety Inventory (STAI) [64]. In addition, we used (5) the Self-transcendence Inventory [33] composed of 10 items to assess Self-transcendence scores, a characteristic of self-decentering. (6) The Five Facets Mindfulness Questionnaire [65] was also used to evaluate mindfulness abilities. A total score was calculated for each participant for all questionnaires (S1 Table).

## Results

### Perceived health, emotion, self-transcendence and mindfulness

Table 2 presents the mean and standard deviation of scores for patients with PD compared to healthy controls across the different reported factors, and the Student-*t* test values and the Cohen’s d, for the between-groups comparisons. To account for multiple comparisons, *p*-values of Student-t tests were corrected using the Benjamini–Hochberg false discovery rate (FDR) procedure. Results were considered statistically significant when *p*-FDR was < 0.05. Previous analyses showed no significant main effect of age or sex, or significant interaction involving these two factors (all *p* > .10). There was only a trend towards significance for the effect of sex on self-transcendence, *F*(1, 94) = 3.40, *p* = .06, η^2^_*p*_ = .04, suggesting that the self-transcendence levels tended to be higher in women (M = 47.00, SE = 2.38) than in men (M = 41.96, SE = 1.67). There was also a significant main effect of sex, *F*(1, 97) = 4.43, *p* = .04, η^2^_*p*_  = .04, and age, *F*(1, 97) = 4.47, *p* = .04, η^2^_*p*_  = .04, for mindfulness. This indicates that mindfulness abilities were higher in men (M = 137.31, SE = 2.15) than in women (M = 126.56, SE = 3.01) and decreased with age.

**Table 2:** Mean scores and standard deviation for the scores of perceived health, affects, self-transcendence and mindfulness for Parkinson Disease (PD) participants and age- and sex-matched control participants, as well as the results of statistical analyses of between-groups comparisons (student t-test, p-FDR, Cohen’s d, and 95% Confidence Interval (lower, upper)).

	PD		Control		t	p FDR^1^	d	95% CI	
	M	SD	M	SD				Lower	Uper
Perceived health	4.04	1.19	5.06	1.09	-4.64	.005	-.89	-1.287	-.495
General Well-being	4.11	1.32	4.77	1.32	-2.61	.01	-.50	-.884	-.117
General happiness	4.31	1.54	4.67	1.34	-1.30	.11	-.25	-.632	.129
Serenity	4.20	1.58	4.79	1.35	-2.04	.03	-.40	-.781	-.012
Stable happiness	59.75	15.82	64.59	13.96	-1.65	.06	-.32	-.708	.063
Fluctuant happiness	36.56	14.14	27.59	10.56	3.63	.005	.71	.314	1.109
Depression	5.80	4.84	3.62	3.76	2.61	.01	.50	.118	.885
Anxiety	42.22	13.05	36.66	10.50	2.38	.02	.47	.077	.857
Self-Transcendence	44.45	11.83	42.47	15.34	.74	.23	.15	-.239	.529
Mindfulness	126.23	19.15	136.25	16.32	-2.92	.007	-.56	-.945	-.175

^1^p-FDR: False discovery rate (FDR) correction using the Benjamini–Hochberg procedure to control for multiple comparisons.

The results presented in Table 2 indicate major differences in perceived affects between the groups. Participants with PD reported poorer health than those in the control group. They also experienced a lower level of well-being and less serenity. They were indeed more depressed and anxious. Nevertheless, their reported level of happiness was not significantly lower, but they did exhibit significantly greater fluctuations in happiness compared to controls. In addition, they showed lower levels of mindfulness. In contrast, self-transcendence scores were similar between the PD participants and those in the control group.

### Passage-of-time judgments

The mean scores for the different types of PoT judgments are presented in Table 3. Previous statistical analyses showed no significant effect of age and sex (all *p* > .10). The effect of sex only approached significance for temporal judgments involving short periods, suggesting that the passage of time in daily life tended to be experienced as faster for men than for women: i.e., present, *F*(1, 94) = 2.97, *p* = .08, η^2^_*p*_  = .03 (M_women_ = 5.43, SE = 0.24; M_men_ = 5.67, SE = 0.17), week, *F*(1, 94) = 3.42, *p* = .07, η^2^_*p*_  = .04 (M_women_ = 5.43, SE = 0.24; M_men_ = 5.67, SE = 0.17), year, *F*(1, 94) = 3.63, *p* = .06, η^2^_*p*_  = .04 (M_women_ = 5.44, SE = 0.25; M_men_ = 5.61, SE = 0.17).

**Table 3:** Mean score and standard deviation of PoT judgments for short (present, week, month, year) and longer periods of life (now compared to 5 years ago, to before and with aging) for Parkinson’s Disease (PD) participants and age- and sex-matched control participants. The results of statistical analyses for the between-groups comparisons were also reported (Student t-test, p FDR, Cohen’s d, 95% Confidence Interval, lower and upper).

	PD		Control		t	*P*	d	95% CI	
PoT	M	SD	M	SD				Lower	Uper
Present	5.44	1.62	5.73	1.09	-1.034	.15	-.202	-.585	.18
Week	5.34	1.56	5.41	1.40	-.248	.40	-.049	-.433	.34
Month	5.43	1.59	5.44	1.33	-.029	.49	-.006	-.388	.38
Years	5.70	1.41	5.50	1.29	.75	.23	.147	-.237	.53
5 years ago	4.98	1.84	4.92	1.79	.16	.44	.032	-.351	.41
Now before	5.00	1.78	5.16	1.64	-.47	.32	-.092	-.476	.29
With aging	5.32	1.81	5.42	1.76	-.294	.39	-.057	-.440	.32
	PD		Control		t	*p FDR*	d	95% CI	
PoT	M	SD	M	SD				Lower	Uper
Present	5.44	1.62	5.73	1.09	-1.034	1.0	-.202	-.585	.18
Week	5.34	1.56	5.41	1.40	-.248	.56	-.049	-.433	.34
Month	5.43	1.59	5.44	1.33	-.029	.49	-.006	-.388	.38
Years	5.70	1.41	5.50	1.29	.75	.81	.147	-.237	.53
5 years ago	4.98	1.84	4.92	1.79	.16	.51	.032	-.351	.41
Now before	5.00	1.78	5.16	1.64	-.47	.75	-.092	-.476	.29
With aging	5.32	1.81	5.42	1.76	-.294	.68	-.057	-.440	.32

Regarding PoT judgments, there was no significant group effect for any of the temporal judgments whether related to short or long periods of life (all *p* > .10). In our study, we were therefore unable to detect that Parkinson’s disease itself has a direct impact on the awareness of the passage of time. Therefore, we investigated whether affect-related factors, which distinguished PD participants from control participants, contributed to inter-individual differences in PoT judgments.

### Relationships between passage-of-time judgments, perceived health, emotion, self-transcendence and mindfulness

Table 4 presents the correlations between the different PoT judgments and perceived health, emotion, self-transcendence and mindfulness-related factors.

**Table 4:** Matrix of correlations between the passage-of-time judgments for short periods (present, week, month, year) and longer periods of life (5 years ago, now-before, with aging), and perceived health, emotion, self-transcendence and mindfulness.

	Present	Week	Month	Year	5 y.	N-bef	Aging	1	2	3	4	5	6	7	8	9
1.Health	.27^**^	.20^*^	.25^*^	.17	.14	.25^*^	.15									
2.Well-being	.35^**^	.32^**^	.34^**^	.24^*^	.22^*^	.28^**^	.24^*^	.73^**^								
3.Happiness	.41^**^	.35^**^	.32^**^	.26^**^	.28^**^	.29^**^	.27^**^	.61^**^	.76^**^							
4.Serenity	.38^**^	.27^**^	.29^**^	.24^*^	.24^*^	.25^*^	.22^*^	.60^**^	.74^**^	.82^**^						
5.Stable hap.	.37^**^	.32^**^	.30^**^	.21^*^	.17	.21^*^	.19	.43^**^	.59^**^	.66^**^	.77^**^					
6.Fluct. hap.	-.18	-.20^*^	-.20^*^	-.18	-.11	-.12	-.09	-.41^**^	-.43^**^	-.35^**^	-.42^**^	-.30^**^				
7.Depression	-.20^*^	-.24^*^	-.18	-.20^*^	-.12	-.13	-.12	-.46^**^	-.49^**^	-.48^**^	-.51^**^	-.55^**^	.59^**^			
8.Anxiety	-.29^**^	-.28^**^	-.25^*^	-.24^*^	-.15	-.17	-.13	-.40^**^	-.53^**^	-.52^**^	-.62^**^	-.66^**^	.59^**^	.62^**^		
9.Self-Trans.	.16	.09	.12	.09	.30^**^	.28^**^	.30^**^	-.01	.15	.23^*^	.23^*^	.34^**^	.05	-.05	-.23^*^	
10.Mindful.	.13	.16	.18	.07	.05	.09	.10	.27^**^	.26^**^	.24^*^	.43^**^	.49^**^	-.54^**^	-.47^**^	-.58^**^	.18

An inspection of Table 4 reveals distinct patterns of correlations between PoT judgments for short periods (present, week, month, year) and those for longer periods of life (5 years ago, now compared to before, with aging), although mindfulness was not correlated with any PoT judgments (*p* > .05).

**Fig 1:**
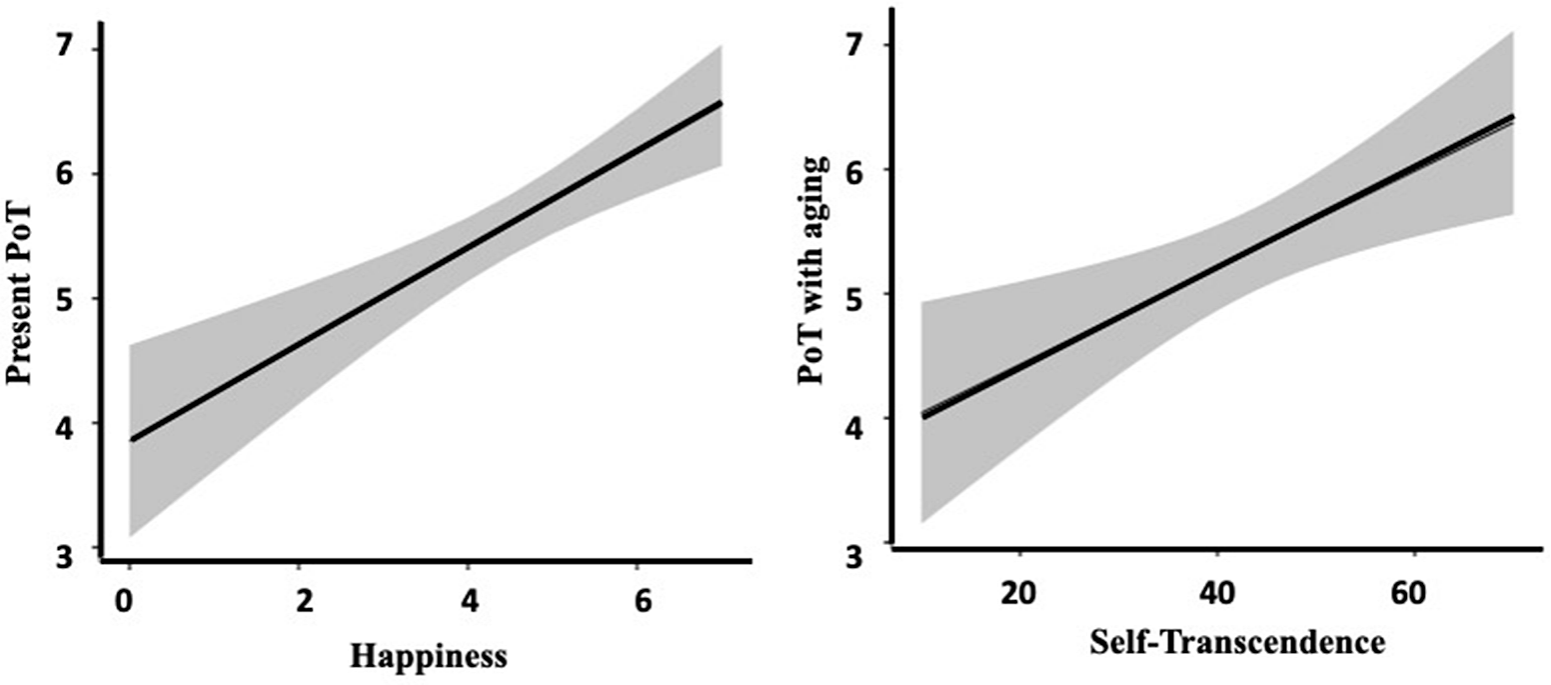
Different predictors for judgments of the passage of time over short and long periods. Happiness is the significant predictor of present passage-of-time judgment (present PoT): the higher the level of happiness, the faster time seems to pass in the present. Self-transcendence is the significant predictor of PoT over long periods, in particular with aging (PoT with aging): the more selfless individuals were, the more they felt that the time had passed faster with aging.

For short periods (present, week, month, year), PoT judgments were not significantly correlated with self-transcendence (all *p* > .05). In contrast, consistent and significant correlations were observed between PoT judgments and factors related to perceived health and emotions. For example, the present PoT scores were significantly correlated with perceived health, *r*  = .27, well-being, *r*  = .35, happiness, *r* = .41, serenity, *r* =.38, stable happiness, *r* = .37, depression, *r* = −.20, and anxiety, *r* = −.29 (all *p* < .01). Perceived health was also strongly correlated with emotion-related factors (e.g., happiness, *r* = .61, *p* < .001). Consequently, to identify the best predictor of PoT judgments (whether perceived health or emotions), we ran a linear regression model for each PoT judgment (present, week, month, year) as the dependent variable, using both perceived health and one of the significant emotion-related factors as independent variables. It should be noted that the emotional factors examined in our study showed a strong correlation with one another (e.g., happiness *vs* well-being, *r* = .76, serenity, *r* = .82, stable happiness, *r* = .66, depression, *r* = −.48, or anxiety *r* = −.52, all *p* < .01). For the model including happiness, which consistently proved to be the factor most strongly correlated with PoT judgments over short periods (present, *r* = .41, week, *r* = .35, month, *r* = .32, year, *r* = .26, all *p* < .01), we found that only happiness remained a significant predictor of the present PoT judgment, B = 0.374, SE = 0.106, Beta = 0.40, *t* = 3.534, *p* < .001, 95% CI [0.163; 0.584], while perceived health lost its predictive power, B = 0.023, SE = 0.121, Be*t*a = 0.021, *t* = 0.189, *p* = .85, 95% CI [−0.218; 0.264]. This finding was systematically verified for happiness with the other *t*ime periods (week, month, year), as well as for other emotional factors and different PoT judgments. Therefore, the higher the level of afflictive affects, the slower the passage of time was experienced and, conversely, the higher the level of positive emotions (e.g., happiness), the faster the passage of time is perceived to be (Fig 1).

For long periods of life (5 years ago, now-before, with aging), there were consistent correlations between PoT judgments and the self-transcendence scores (*r* = 0.30, *r* = 0.28, *r* = 0.30, respectively, all *p* < .01). However, some emotion-related factors (well-being, happiness, serenity) were also significantly correlated with these PoT judgments for long periods. In addition, the more selfless the participants were, the happier and more serene they were, with a more stable level of happiness (*r* = 0.23, *r* = 0.23, *r* = 0.34, respectively, *p* < .01). Therefore, we included in the same linear regression model the self-transcendence factor and the significantly correlated emotional factors (well-being, happiness, serenity) for each PoT judgment (5 years, now-before, with aging) (Table 5). These models revealed that only the self-transcendence factor remained a significant predictor of these PoT judgments (all *p* < .05), while the emotion-related factors were no longer significant (*p* > .05). Therefore, the more selfless individuals were, the more they felt that the time had passed faster with aging (Fig 1), now compared to before, and over the past 5 years.

**Table 5:** Linear regression models for PoT judgments (now compared to 5 years ago, now compared to before, with aging) with the self-transcendence factor, and the significant correlated emotion-related factor (well-being, happiness, serenity).

	Judgment of the passage of time now compared to 5 years ago									
						95% CI				
Model	B	SE	Beta	t	*p*	Lower	Upper	Part	VIF	R^2^
Self-Transcendence	.034	.013	.257	2.643	.010	.009	.060	.249	1.064	
Well-being	.039	.198	.031	.200	.842	-.353	.432	.019	2.668	
Happiness	.272	.219	.227	1.246	.216	-.161	.706	.117	3.726	
Serenity	-.046	.204	-.039	-.224	.823	-.451	.359	-.02	3.419	.14^**^
	Judgment of the passage of time now compared to before									
						95% CI				
Model	B	SE	Beta	t	*p*	Lower	Upper	Part	VIF	R^2^
Self-Transcendence	.029	.012	.231	2.369	.020	.005	.052	.224	1.064	
Well-being	.192	.184	.161	1.044	.299	-.173	.558	.099	2.664	
Happiness	.202	.204	.181	.990	.325	-.203	.607	.094	3.737	
Serenity	-.094	.192	-.086	-.489	.626	-.474	.287	-.05	3.437	.14^**^
	Judgment of the passage of time with aging									
						95% CI				
Model	B	SE	Beta	t	*p*	Lower	Upper	Part	VIF	R^2^
Self-Transcendence	.034	.013	.256	2.633	.010	.008	.059	.249	1.064	
Well-being	.130	.195	.102	.665	.508	-.257	.517	.063	2.668	
Happiness	.232	.215	.196	1.075	.285	-.196	.659	.102	3.726	
Serenity	-.095	.201	-.083	-.473	.638	-.494	.304	-.05	3.419	.14^**^

### Further examination of the relationship between passage-of-time judgments and individual items of the self-transcendence scale

The results clearly show a significant relationship between PoT and self-transcendence for long periods of time, but not for short periods of time. The Self-transcendence Inventory by Levenson et al. [33] consists of 10 items assessing various aspects of self-transcendence, such as “Material things mean less to me”, “I find more joy in life”, or “I feel a greater state of belonging with both earlier and future generations”. Therefore, to better understand the relationship between PoT for long periods and the self-transcendence dimensions, we examined the correlations between each PoT judgment and the individual items of this scale (Table 6).

**Table 6:** Correlations between the passage-of-time judgments for short periods (present, week, month, years) and longer periods of life (5 years ago, now-before, with aging), and individual responses on each of the 10 items of Levenson’s self-transcendence scale.

	PoT						
	Present	Week	Month	Years	5 years	Now before	With aging
1. My peace of mind is not so easily upset as it used to be.	0.14	0.08	0.09	0.10	0.17	0.16	0.16
2. Material things mean less to me.	−0.02	−0.02	−0.02	0.02	0.12	0.06	0.02
3. I do not become angry as easily.	0.01	−0.06	−0;02	−0.13	0.04	0.00	0.02
4. My sense of self is less dependent on other people and things.	0.05	−0.02	0.01	0.02	0.12	0.17	0.16
5. I feel much more compassionate, even toward my enemies.	0.15	0.11	0.11	0.10	0.22*	0.16	0.23*
6. I find more joy in life.	0.19	0.14	0.17	0.05	0.27**	0.27**	0.16
7. I am more likely to engage in quiet contemplation.	0.12	0.06	0.06	−0.01	0.26**	0.26*	0.29**
8. I feel a greater state of belonging with both earlier and future generations	0.28**	0.16	0.19	0.16	0.29**	0.32**	0.36***
9. I feel that my individual life is a part of a greater whole.	0.04	0.07	0.10	0.07	0.31***	0.28***	0.16
10. I have become less concerned about other people’s opinions of me.	0.16	0.08	0.09	0.18	0.34***	0.32***	0.33***
Self-transcendence (mean score)	0.16	0.09	0.12	0.09	0.30**	0.28**	0.30**

Table 6 confirms the finding that no self-transcendence item was significantly correlated with PoT judgments for short periods (all *p* > .05), except for a single item (item 8, belonging with past and future generations) and the present PoT judgment. In contrast, several items of the scale were significantly correlated with the different PoT judgments for long periods. The items that were systematically correlated with all 3 long-term PoT judgments (5 years, now-before, with aging) were: item 6 (joy in life), item 7 (quiet contemplation), item 8 (belonging with past and future generations), and item 10 (less concerned for others’ opinion). However, the strongest correlation was found for item 8 (mean *r* = .33), in particular for the “PoT with aging” (*r* = .36, *p* < .001), followed closely by item 10 (mean *r* = .33).

## Discussion

In this study, we examined different types of PoT judgments for both short and long time periods in participants with Parkinson’s disease compared to a group of healthy controls. We also assessed several dimensions of psychological functioning to determine which factors influenced PoT judgments.

The results showed no significant effects of age or sex for most of the dimensions examined. A significant effect of sex and age was only observed for mindfulness scores, suggesting that men were more oriented toward mindfulness than women, and that mindfulness decreased with age whether or not the participants were PD patients. Mindfulness refers to a discipline of consciousness in which individuals remain engaged in the present moment while acknowledging and accepting their thoughts and feelings [66]. Several studies have demonstrated that this personality trait, as well as the practice of mindfulness meditation, is associated with higher levels of well-being and happiness, and lower levels of anxiety [67]. Our findings provide additional evidence for the link between mindfulness and well-being in the health domain. Specifically, in our study, higher mindfulness scores were associated with better perceived health, greater happiness and serenity, and lower levels of depression and anxiety. Our results revealed that mindfulness abilities were lower in PD patients than in healthy controls. In addition, participants with Parkinson’s disease reported lower levels of perceived health compared to controls, although the latter, who were also older, did not report particularly high health levels (mean score of 5 out of 7). Lower subjective health evaluations were strongly correlated with lower levels of well-being, happiness, and serenity, and were significantly associated with higher levels of depression and anxiety.

These results are in agreement with numerous studies describing the alteration of several psychological dimensions associated with Parkinson’s disease. Indeed, due to the disruption of the dopaminergic network, this neurodegenerative disorder induces hypervigilance to negative affect [68,69] and difficulties in emotional regulation, which increase the risk of anxiety and depression [50,51,70,71]. Furthermore, interoceptive awareness appears to be altered in PD [46,72], which may contribute to difficulties in emotional regulation. Since mindfulness abilities strongly depend on interoception and attentional mechanisms, it is unsurprising that PD patients exhibit reduced mindfulness skills. The literature on personality-related self-regulation in PD also indicates that apathy is a major non-motor symptom [49,52]. Apathy is characterized by a lack of motivation that affects behavioral initiative but also emotion, i.e., reducing emotional reactivity. Previous studies have reported alterations in fronto-striatal networks associated with apathetic behavior [52]. These neural changes have also been linked to cognitive deficits, particularly attentional deficits [73]. Consequently, both apathy and attention deficits could explain the PD patients’ difficulties in mindfulness.

By contrast, PD patients exhibited similar overall levels of self-transcendence as healthy controls. Self-transcendence is defined as the pursuit of meaning that extends beyond oneself and one’s own needs or interests. As self-transcendence is more tuned by cognitive representations of the self rather than by physiological and interoceptive dimensions, there is actually no particular reason to observe an alteration of this dimension with PD. Indeed, self-transcendence is triggered by meaning seeking and corresponds to an attitude toward the meaning of life that does not depend on illness, but that can be a strong protective factor to cope with life difficulties by reducing the focus on the body and the disease. Therefore, even if PD induces psychological distress in patients, a high level of self-transcendence could help them reduce ruminations about self-projection into a difficult future. In this sense, even if PD patients present a psychological burden linked to afflictive affects, this does not imply that they never experience momentary happiness, as suggested by our data, showing higher scores of fluctuating happiness in PD patients compared to healthy controls, while their levels of stable happiness were comparable. Nonetheless, a persistent issue is that these afflictive emotional states in PD patients often remain undiagnosed and underestimated, even though they considerably impact quality of life.

With regard to PoT judgments, our results showed that PoT judgments did not significantly vary as a function of mindfulness scores, but rather as a function of emotion experienced when judging the passage of time over short periods, and self-transcendence when judging the passage of time over long periods. According to the Contextual Self-Duration Theory, judgments about the passage of time in the present are not based on temporal information processing per se, but rather on awareness of the self within a temporal dynamic [9,10,15]. In line with this theory, our study shows that the significant predictors of inter-individual variations in PoT judgments for short periods (present, week, month, year) were the emotions experienced (well-being, happiness, serenity) rather than scores on self-transcendence related to the narrative self. Specifically, the more individuals experienced afflictive emotional states, the more they reported a slowing of the passage of time. This finding is consistent with previous studies showing that depressed individuals often report a slowing of time, such that “a day feels like a year” [74,75].

In our study, inter-individual differences in emotion-related factors (well-being, happiness, serenity) were likely amplified by the inclusion of people with PD in our sample. This allowed us to clearly support the idea that PoT judgments for short periods stem from introspective analyses of one’s emotional state within a given context (minimal self) [10,15]. Accordingly, when patients with Parkinson’s disease are overwhelmed by negative emotions linked to their perceived poor health, they tend to experience a subjective slowing of time. Given that emotional states are the primary predictors of PoT judgments for short periods, our results suggest that it is not Parkinson’s disease per se, but rather the emotional symptoms and self-related beliefs associated with it, that indirectly shape PoT judgments.

However, the absence of detectable differences between PD and control participants in awareness of the passage of time may constitute a limitation of our study. Future studies should determine whether this result can be replicated or whether it reflects a genuine limitation of the present study, for example, due to the characteristic of the sample studied. Parkinson’s disease is indeed characterized by significant heterogeneity, so that impairments in duration perception among patients with Parkinson’s disease have been a subject of debate, with some studies reporting timing deficits whereas others do not [76]. In addition, we found no significant group differences in PoT judgments, despite the lower interoceptive abilities observed in PD participants in previous studies [46,47]. In addition, it has been shown that individuals with higher interoceptive awareness exhibit greater changes in PoT judgments during tasks [14], suggesting that interoceptive awareness plays a key role in PoT judgments [e.g., 14,17,20]. It is thus possible that our self-reported questionnaire measures lacked sufficient sensitivity. It is also possible that the awareness deficit in Parkinson’s disease associated with underlying brain dysfunction does not directly affect the subjective experience of time. A distinction exists between objective cognitive impairment and subjective experience, as these two domains do not necessarily deteriorate in parallel [77]. This distinction is supported by studies reporting relative preserved metacognitive judgments in PD despite variability in their cognitive and motor symptoms [77,78]. Future studies should therefore replicate our results and investigate the specific role of interoception in potential difference in PoT judgments between PD and control participants. Consequently, the absence of significant differences in PoT between groups in our study should be interpreted with caution.

Unlike the PoT judgments for short periods, it is self-transcendence, rather than emotion-related factors, which has been found to be a significant predictor of PoT judgments for long time periods. However, our results indicated that self-transcendence scores were significantly correlated with reported happiness, consistent with previous research showing that self-transcendence fosters a general sense of happiness [32,79]. It is therefore plausible that the positive emotions associated with this personality trait contribute to the sensation of time accelerating with aging or across extended life periods. However, our regression analyses suggest that happiness lost its predictive power when entered alongside self-transcendence in the same model. Self-transcendence thus emerged as the only significant predictor of PoT judgments for long periods in our study.

Our study is the first to highlight the key role of self-transcendence in PoT judgments over long life spans. To further clarify this link, we examined the correlations between individual items of the self-transcendence scale and PoT judgments. The strongest correlation emerged for item 8, particularly with respect to “PoT with aging,” which reflects a heightened sense of belonging to past and future generations. This finding supports the idea that an expanded consciousness beyond the individual self can reshape the perception of time across the lifespan. Temporal self-continuity, the sense of connection between oneself and both past and future generations, broadens the self across time, thereby reducing the subjective “weight” of the present. This expanded temporal perspective fosters a more coherent life narrative, in which the past, present, and future are integrated, ultimately leading to a retrospective compression of lived time. Item 10 (less concern about other people’s opinions) may also reflect a form of detachment from immediate concerns, reinforcing this broader temporal outlook. Taken together, these findings suggest a holistic perception of time in which life is experienced as part of a wider temporal continuum, giving rise to a subjective acceleration of time across one’s life trajectory. Previous studies have shown that self-transcendence, by supporting the feeling of being connected to an intergenerational history or to humanity, could act as a defensive strategy against death anxiety [38,65,80–82].

Finally, the other items that correlated with PoT judgments over long periods were related to personality traits such as “quiet contemplation,” “joy in life,” and “greater compassion.” Although several previous studies have shown the effect of emotions on present PoT, no studies have demonstrated this emotional effect on PoT over long time periods. Our findings suggest that a form of wisdom associated with a positive mood (joy in life, contemplation) may retrospectively compress the perception of one’s lifetime. However, our results indicate that participants with higher levels of self-transcendence tended to feel that time passed more quickly, regardless of the emotional states linked to their “emotional” personality traits. In addition, there is a strong relationship between wisdom, temporal self-continuity, and efficient coping strategies. Further research is needed to disentangle these different mechanisms and to better understand their respective impacts on PoT judgments. Our study also suggests a key role of generational belonging. But does this feeling accelerate time perception, or does reflecting on the passage of time strengthen this sense of belonging? Does this self-reported perception reflect emotional bonds (e.g., nostalgia) or cognitive beliefs (e.g., belief in paradise or reincarnation)? Investigating how cultural belief systems influence different types of PoT judgments could provide valuable insights. Finally, while PoT judgments over long time periods have often been considered stable beliefs [11,13] or even illusions [83], our study suggests that such judgments may vary depending on the semantic temporal reference used and the degree of temporal self-continuity. The representation of self is thus at the heart of different types of PoT judgments [84].

In conclusion, our findings revealed no difference in PoT judgments between participants with Parkinson’s disease and control participants. This finding needs to be confirmed by further studies, as self-report measures may lack sensitivity. Nevertheless, this lack of difference between groups led us to suggest that it is not Parkinson’s disease per se that directly affects PoT judgments in daily life, but rather the emotional symptoms associated with it (reduced perceived health, well-being, happiness, serenity). These affective disturbances are linked to a subjective experience of daily time passing more slowly. In contrast, PoT judgments concerning long periods of life, such as those related to aging, appear to be associated with the narrative self and self-transcendence. Our study suggests that a holistic perception of time, supported by a sense of temporal self-continuity, increases the feeling that time passes more quickly with aging.

## Supporting information

S1 Table Data table. Basic data used in the statistical analyses of the study: Monier et al. (2026). Parkinson’s disease and awareness of the passage of time: Perceived health, emotion, and self-transcendence.
(XLSX)
